# Coronavirus disease 2019 in Botswana: Contributions from family physicians

**DOI:** 10.4102/phcfm.v12i1.2497

**Published:** 2020-07-09

**Authors:** Keneilwe Motlhatlhedi, Yaone Bogatsu, Koketso Maotwe, Billy Tsima

**Affiliations:** 1 Department of Family Medicine and Public Health, Faculty of Medicine, University of Botswana, Maun, Botswana; 2 Department of Family Medicine and Public Health, Faculty of Medicine, University of Botswana, Gaborone, Botswana; 3 Private Practice, Orapa, Botswana

**Keywords:** COVID-19, Botswana, family medicine, primary care, contributions

## Abstract

Coronavirus disease 2019 (COVID-19), the respiratory disease caused by the virus now called as SARS-CoV-2 and first identified in Wuhan, China, has spread to all regions of the world. At the time of this write-up, over 5.1 million people had been infected by the virus globally. The World Health Organization estimates that in Africa over 5 million people would need hospital admission during the course of the pandemic. Interventions to prevent the disease include social distancing and nationwide lockdowns, which, whilst necessary, have had negative effects not only on the economic status of many but also on primary care and especially the management of chronic illnesses. There are opportunities for primary care physicians to continue learning, lend humanitarian aid and provide the needed care in this context. Social media has promising applications in this rapidly changing context.

## Introduction

The respiratory disease caused by the virus, now called SARS-CoV-2, and first identified in Wuhan, China, has spread to all regions of the world.^1^ The disease caused by the virus is now referred to as Coronavirus disease 2019 (COVID-19). At the time of this write-up, over 5 million people had been infected by the virus globally, with more than 330 000 deaths.^2^ The World Health Organization has estimated that if containment efforts are failed, Africa would have a prolonged outbreak, with over 44 million people being at risk of infection and 5.5 million of these requiring hospital admission.^3^ In response to this rapidly spreading disease, many countries closed their borders and adopted varying degrees of social isolation strategies.

Botswana, a southern African country, with an estimated population of 2.5 million, reported its first case of COVID-19 on 30 March 2020. At the time of writing this report, there were 29 confirmed cases and one death in the country. Botswana closed its borders on 24 March 2020 and introduced a mandatory quarantine of 14 days for all those entering the country. At the same time, extreme social distancing with closing of schools and non-essential services was implemented. A state of emergency (SOE) was declared in Botswana on 02 April 2020, further restricting non-essential movements that could facilitate the spread of COVID-19. The COVID-19 response is coordinated from a national level and each district implements case management, infection control and prevention and logistics strategies that are adapted for the district from the national strategy. A national COVID relief fund has been set up to raise funds to support those who lost their earning capacity and to augment government efforts during this pandemic.

Primary care services were re-structured to reduce over-crowding in health facilities. The Ministry of Health and Wellness (MoHW) recommended extension of review periods for stable chronic care patients and allowed for the chronic medication refills at the pharmacy without need for prior doctor’s consultation. Family physician-led facilities have developed implementation plans dichotomising chronic care patients into those who would be reviewed and those who would get extended medication refills. The review of acute care patients continues as before. Maternal and child healthcare services have been restricted to immunisations and care for children with malnutrition, antenatal visits with extended intervals and block booking, except for those in the third trimester or with high-risk pregnancies. Some healthcare workers have been re-deployed to the COVID-19 isolation centres, local task forces included post-graduate residents in family medicine and shift work has been introduced in some facilities to reduce crowding.

Further infection control measures include screening for risk of COVID-19 at the entrances of many of the facilities, private or public, and maintaining social distancing among patients while seating in the waiting areas. All healthcare workers are provided with N95 respirator masks, gloves and either soap and water or hand sanitisers for hand hygiene. Public health facilities have makeshift handwashing points set up for patients.

## Family medicine in the pandemic

**FIGURE 1:**
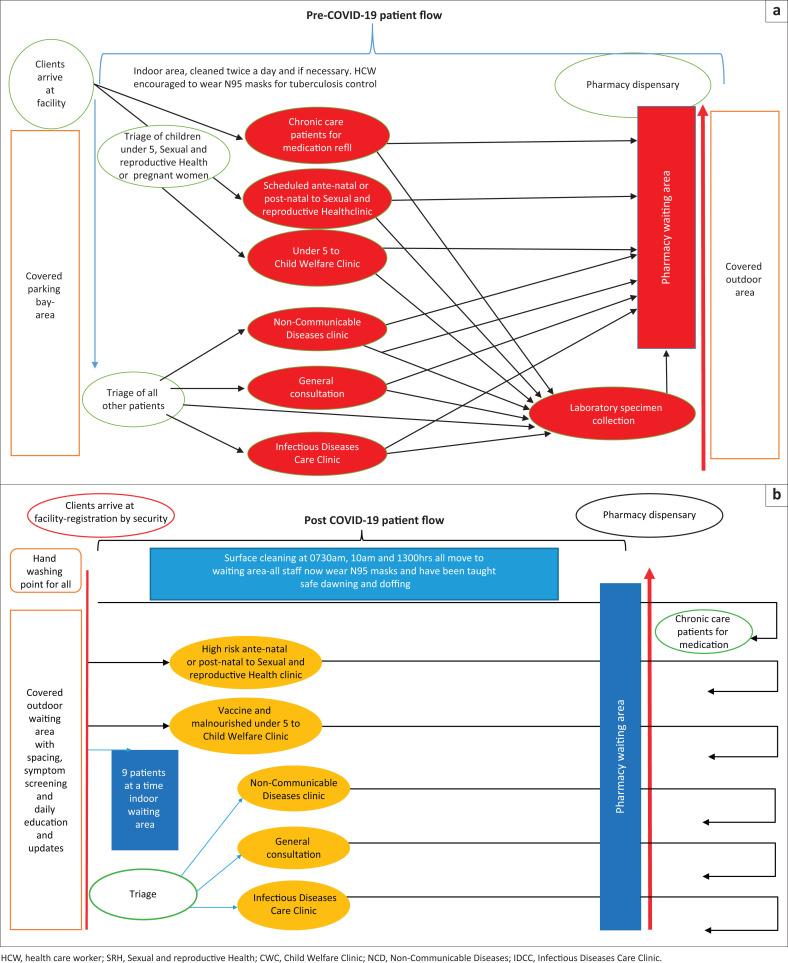
Patient flow diagram from a facility in the North-West District: (a) Pre-COVID-19, (b) Post- COVID-19.

Following the emergence of COVID-19, family physicians and other primary care clinicians in Botswana have been active in local guideline development and training of district healthcare workers. They are involved in suspect screening at the main isolation centres as well as in the districts. In addition, family physicians who led primary care facilities have been instrumental in helping to design patient flow by working with other primary care team members. An example of patient flow adjustments from a facility in the North-West District is presented in Figure 1.

The implementation of social distancing measures has increased the need to adopt online communication platforms. Prior to the pandemic, family physicians and general practitioners in Botswana had been using WhatsApp groups to share official communication from the Ministry of Health and clinical updates. This use of social media has expanded to include online webinars, dissemination of COVID-related information from various sources and from the national coordination centre as well as for sharing innovation in patient care. Based on our observation, social media has facilitated faster dissemination of information especially to practitioners in rural areas. Furthermore, social media use has been extended to patient care. Some private health practitioners have started using telehealth consultations for their patients. However, in the public health sector, implementing telehealth is challenging because of the limited network connectivity and a largely patient-held medical record system. We believe that this highlights the need to adopt information technology in primary care, not only for the current situation but also as a tool to enhance patient access and coordination of care.

The lockdown has limited the ability of community members to fend for themselves. This has disproportionately affected those members of society who are already vulnerable. Family physicians are involved in caring for the less privileged members of society through their prior engagement with various local organisations. Some family physicians are members of charity organisations, whilst others, by virtue of being family physicians in the community or because of their own accord, have been working with community-based groups. Additionally, family physicians, especially those in the more remote areas, are on advisory boards of local businesses and community fora such as churches. By virtue of their medical background, some of these family physicians are engaged in teaching communities about COVID-19 and its prevention and risk reduction. This is a key role that family physicians play as active members of the communities in which they live.

## Conclusion

The role of family physicians in Botswana’s response to COVID-19 has been a direct involvement not only in infection control and disease prevention measures, case identification and information dissemination but also in continuing medical care, especially for chronic care patients at the primary care level.
